# High ratio of monocytes to high-density lipoprotein is associated with hemorrhagic transformation in acute ischemic stroke patients on intravenous thrombolysis

**DOI:** 10.3389/fnagi.2022.977332

**Published:** 2022-08-16

**Authors:** Lingfan Xia, Tong Xu, Zhenxiang Zhan, Yucong Wu, Ye Xu, Yungang Cao, Zhao Han

**Affiliations:** Department of Neurology, The Second Affiliated Hospital and Yuying Children’s Hospital of Wenzhou Medical University, Wenzhou, China

**Keywords:** high-density lipoprotein, hemorrhagic transformation, thrombolysis, recombinant tissue plasminogen activator, acute ischemic stroke, monocyte

## Abstract

**Background:**

Hemorrhagic transformation (HT) is a frequent, serious complication in acute ischemic stroke patients on intravenous thrombolysis. Here we investigated whether risk of HT is associated with the ratio of monocyte count to high-density lipoprotein level (MHR).

**Materials and methods:**

Medical records were retrospectively examined for consecutive patients with acute ischemic stroke who received thrombolytic therapy. HT was diagnosed by computed tomography at 24–36 h after therapy. Potential association between MHR and HT was examined using logistic regression.

**Results:**

A total of 340 patients were analyzed, and their median MHR was 0.44 (0.31–0.59). MHR was higher in the 51 patients (15.0%) with HT than in those who did not suffer HT (0.53 vs. 0.42, *P* = 0.001). Multivariate logistic regression showed that, after adjusting for potential confounders, MHR was an independent risk factor for HT (OR 7.50, 95% CI 1.64 to 34.35, *P* = 0.009). Risk of HT was significantly higher among patients whose MHR fell in the third quartile (0.42–0.53) and the fourth quartile (> 0.53) than among those with MHR in the first quartile (< 0.31; OR 3.53, 95% CI 1.11 to 11.20, *P* = 0.032; OR 4.79, 95% CI 1.49 to 15.42, *P* = 0.009).

**Conclusion:**

High MHR may be independently associated with higher risk of HT in patients with acute ischemic stroke on intravenous thrombolysis.

## Introduction

Intravenous thrombolysis using recombinant tissue plasminogen activator within 4.5 h of stroke onset can significantly reduce morbidity and mortality (Hacke et al., 2008). However, such thrombolytic therapy can increase risk of hemorrhagic transformation (HT), which occurs in 10–43% of patients (Yaghi et al., 2017). HT can lead to reduced cognitive function in the long term and even death (Dzialowski et al., 2007; He et al., 2022). Understanding more about how HT occurs and its risk factors may help identify at-risk patients and formulate treatment plans to improve their prognosis.

Hemorrhagic transformation may be caused by post-ischemic inflammatory responses that disrupt the blood-brain barrier (Jickling et al., 2014). Monocytes, as the major group of inflammatory cells, infiltrate the ischemic area, where they secrete pro-inflammatory cytokines (Enciu et al., 2013) that exacerbate brain injury (Jin et al., 2010; Kim and Cho, 2016). High-density lipoprotein (HDL) can counteract this inflammation and thereby protect the blood-brain barrier by preventing monocytes from attaching to endothelial cells and by inhibiting oxidation of low-density lipoprotein (Murphy et al., 2008). Thus, we wondered whether the ratio of monocyte count to HDL level (MHR) might be useful as a prognostic indicator in patients with acute ischemic stroke on intravenous thrombolysis. Indeed, the MHR has already shown prognostic value in different types of cardiovascular diseases (Karataş et al., 2016; Zhang et al., 2020; Jiang et al., 2022), stroke-associated pneumonia (Sun et al., 2021) and acute ischemic stroke (Liu et al., 2020).

Here we explored the potential relationship between MHR and HT specifically in patients on intravenous thrombolysis.

## Materials and methods

### Study population

This study retrospectively analyzed data from patients with acute ischemic stroke who were admitted to the Department of Neurology at the Second Affiliated Hospital and Yuying Children’s Hospital of Wenzhou Medical University (Wenzhou, China) from July 2014 to May 2021 and who received intravenous recombinant tissue plasminogen activator within 4.5 h of stroke onset. Patients had to be at least 18°years old. Patients were excluded if computed tomography was not performed within 36 h after intravenous thrombolysis; if essential data were unavailable, especially monocyte count or HDL level within 24 h after admission; or if patients had severe infection or immune disorder.

Acute ischemic stroke was diagnosed based on World Health Organization criteria (Mendelson and Prabhakaran, 2021). Patients were given recombinant tissue plasminogen activator at 0.9 mg/kg (up to 90 mg total) on arrival in the emergency room.

This study protocol was approved by the Ethics Committee of the Second Affiliated Hospital and Yuying Children’s Hospital of Wenzhou Medical University. The Committee waived the requirement for informed consent since, at the time of treatment, patients or their legal guardians consented to the analysis and publication of their anonymized medical data for research purposes.

### Clinical and laboratory tests

Trained neurologists assessed stroke severity at baseline using the National Institutes of Health Stroke Scale (NIHSS), and they identified possible stroke etiologies according to the TOAST classification.

Venous blood was sampled from all patients at 24 h after thrombolytic treatment and analyzed using an automatic hematology analyzer (XE-5000, Sysmex, Kobe, Japan) and an automated biochemical analyzer (AU5800, Beckman Coulter, Brea, CA, United States). Monocyte count (× 10^9^/L) was divided by HDL level (mmol/L), both determined in the same blood sample, in order to calculate MHR.

### Diagnosis of hemorrhagic transformation

All patients underwent computed tomography of the brain on admission and within 24–36 h after intravenous thrombolysis. HT was defined as intracranial hemorrhage that was not detected on the initial tomography but appeared on follow-up tomography. It was classified according to ECASS criteria as hemorrhagic infarction HI1 or HI2, involving punctate hemorrhage in the infarction area; or parenchymal hematoma PH1 or PH2, involving a distinct hemorrhage area with or without mass effect (Hacke et al., 1995). Researchers diagnosed HT based purely on computed tomography; they were blinded to the patients’ other clinical data.

### Additional data collected

Data were extracted from medical records about age and sex; vascular risk factors, including hypertension, diabetes, hyperlipidemia, coronary heart disease, current smoking, and current alcohol consumption; baseline clinical data, including systolic blood pressure, diastolic blood pressure, blood glucose level, interval between stroke onset and treatment, current antiplatelet therapy; and bridging therapy.

### Statistical analysis

Continuous variables were expressed as mean ± standard deviation (SD) or median (interquartile range, IQR), and inter-group differences were assessed for significance using Student’s *t* test or the Mann–Whitney *U* test. Categorical variables were expressed as frequency (percentage), and inter-group differences were assessed using Pearson’s chi-squared test or Fisher’s exact test.

Crude model analyses and multivariate logistic regression was conducted to identify independent risk factors for HT. Only MHR was included in the model because of the close relationship between the ratio and its components, monocyte count and HDL level. MHR was entered into the model either as a continuous variable, in which case the odds ratio (OR) for HT was calculated in terms of each 1-SD increase; or as a quartile variable, in which case OR was calculated using the first quartile as reference. The models were adjusted for variables that were associated with *P* < 0.1 in the univariate analysis or that had already been linked to HT in the literature.

We calculated areas under receiver operating characteristic curves (AUCs) to assess the ability of MHR to identify patients who experience HT. The optimal cut-off MHR was determined to be the value that gave the greatest sum of specificity and sensitivity.

All statistical analyses were performed using SPSS 23.0 for Windows (IBM, Chicago, IL, United States). Results associated with two-tailed *P* < 0.05 were considered statistically significant.

## Results

### Patient characteristics at baseline

**FIGURE 1:**
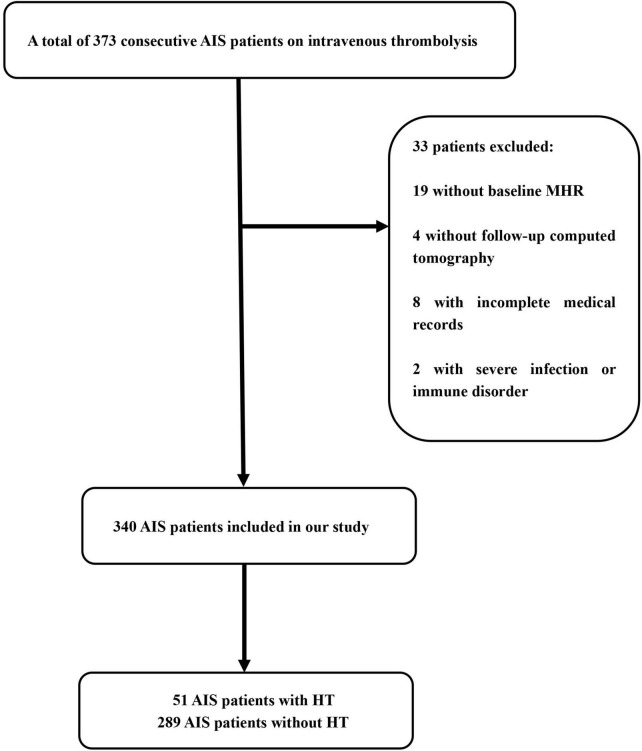
Flow diagram of patient enrollment. AIS, acute ischemic stroke; HT, hemorrhagic transformation; MHR, monocyte to high-density lipoprotein ratio.

Of the 373 patients with acute ischemic stroke who were treated intravenously with recombinant tissue plasminogen activator during our enrollment period, we excluded 19 with no baseline MHR, four with no follow-up computed tomography, eight with otherwise incomplete medical records, and two with severe infection or immune disorder. In the end, our analysis included 340 patients (65.0% male) with a mean age of 69.5 ± 13.5°years (Figure 1 and Table 1).

**TABLE 1:** Characteristics of the entire study population (*n* = 340).

Characteristic	Value
**Demographics**	
Age (years)	69.5 13.5
Female	119 (35.0)
**Medical history**	
Hypertension	264 (77.6)
Diabetes mellitus	113 (33.2)
Hyperlipidemia	124 (36.5)
Atrial fibrillation	103 (30.3)
Previous stroke history	44 (12.8)
Coronary heart disease	32 (9.4)
**Medication history**	
Antiplatelets	44 (12.9)
**Cardiovascular risk factors**	
Current smoking	76 (22.4)
Current drinking	67 (19.7)
Clinical features NIHSS on admission	8 (4–14)
Baseline SBP (mmHg)	159.0 1.25
Baseline DBP (mmHg)	88.4 0.87
Baseline blood glucose (mmol/L)	7.0 (6.02–8.98)
White blood cell count (× 10^9^/L)	7.9 (6.21–10.31)
Monocyte count (× 10^9^/L)	0.45 (0.34–0.59)
Triglycerides (mmol/L)	1.23 (0.92–1.78)
Total cholesterol (mmol/L)	4.33 (3.63–5.13)
HDL (mmol/L)	1.05 (0.86–1.27)
MHR	0.44 (0.31–0.59)
ONT (min)	170 (125–210)
Platelet count (× 10^9^/L)	191 (162–232)
International normalized ratio	1.03 (0.98–1.09)
APTT (sec)	34.2 (31.1–37.1)
Bridge therapy	38 (11.2)
**TOAST classification**	
Large–artery atherosclerosis	131 (38.5)
Small–artery occlusion	88 (25.9)
Cardioembolic	76 (22.4)
Other etiology	2 (0.6)
Undetermined etiology	43 (12.6)

Values are n (%), mean SD, or median (interquartile range).

APTT, activated partial thromboplastin time; DBP, diastolic blood pressure; HDL, high-density lipoprotein cholesterol; MHR, monocytes to high-density lipoprotein; NIHSS, National Institutes of Health Stroke Scale; ONT, onset-to-treatment time; SBP, systolic blood pressure.

Of these patients, 51 (15.0%) experienced HT, which was classified as HI1 in 18 (5.3%), HI2 in 16 (4.7%), PH1 in 8 (2.4%), or PH2 in 9 (2.6%). HT was associated with significantly higher incidence of atrial fibrillation (51.0% vs. 26.6%, *P* < 0.001), NIHSS score at admission (17 vs. 4, *P* < 0.001), white blood cell count (10.72 vs. 7.66, *P* < 0.001), monocyte count (0.57 vs. 0.43, *P* < 0.001), and international normalized ratio (1.06 vs. 1.03, *P* = 0.012) (Table 2). Bridging therapy was also significantly more frequent among patients with HT (19.6% vs. 9.7%, *P* = 0.038). Crude models for HT also showed that NIHSS, atrial fibrillation, white blood cell count, monocyte count, bridging therapy might be associated with HT (*P* < 0.05; Table 3).

**TABLE 2:** Comparison of characteristics between patients who experienced hemorrhagic transformation (HT) or not.

Characteristic	No HT (*n* = 289)	HT (*n* = 51)	*P*
**Demographics**			
Age (years)	69.3 ± 13.5	71.5 ± 13.5	0.278
Female	99 (34.3)	20 (39.3)	0.494
**Medical history**			
Hypertension	222 (76.8)	42 (82.4)	0.382
Diabetes mellitus	98 (33.9)	19 (37.3)	0.643
Hyperlipidemia	107 (37.0)	17 (33.3)	0.614
Atrial fibrillation	77 (26.6)	26 (51.0)	< 0.001*
Coronary heart disease	24 (8.3)	8 (15.7)	0.116
**Medication history**			
Antiplatelets	35 (12.1)	9 (17.6)	0.277
**Cardiovascular risk factors**			
Current smoking	68 (23.5)	8 (15.7)	0.215
Current drinking	57 (19.7)	10 (19.6)	0.985
**Clinical features**			
NIHSS on admission	7 (4–13)	12 (7–18)	< 0.001*
Baseline SBP (mmHg)	158.7 ± 21.9	160.9 ± 28.6	0.623
Baseline DBP (mmHg)	88.0 ± 16.2	90.9 ± 15.5	0.224
Baseline blood glucose (mmol/L)	7.02 (6.00–8.92)	7.25 (6.34–9.38)	0.307
White blood cell count (× 109/L)	7.66 (5.97–9.90)	10.72 (7.73–11.97)	< 0.001*
Monocyte count (× 109/L)	0.43 (0.33–0.56)	0.57 (0.43–0.74)	< 0.001*
Total triglycerides (mmol/L)	1.12 (0.80–1.36)	1.29 (0.94–1.79)	0.062
Total cholesterol (mmol/L)	4.41 (3.65–5.14)	4.05 (3.58–4.61)	0.178
HDL (mmol/L)	1.06 (0.85–1.27)	1.05 (0.93–1.27)	0.601
MHR	0.42 (0.29–0.57)	0.53 (0.42–0.69)	0.001*
ONT (min)	165 (114–198)	170 (127–213)	0.133
Platelet count (× 10^9^/L)	191 (164–234)	184 (156–217)	0.099
International normalized ratio	1.03 (0.98–1.09)	1.06 (1.01–1.11)	0.012*
APTT (sec)	34.3 (31.0–37.1)	33.8 (31.6–37.9)	0.924
Bridge therapy	28 (9.7)	10 (19.6)	0.038*
TOAST classification			0.01*
Large–artery atherosclerosis	111 (38.4)	20 (39.2)	
Small–artery occlusion	72 (24.9)	4 (7.8)	
Cardioembolic	67 (23.2)	21 (41.2)	
Other etiology	1 (0.3)	1 (2.0)	
Undetermined etiology	38 (13.1)	5 (9.8)	

Values are n (%), mean SD, or median (interquartile range).

APTT, activated partial thromboplastin time; DBP, diastolic blood pressure; HDL, high-density lipoprotein cholesterol; HT, hemorrhagic transformation; MHR, monocytes to high-density lipoprotein; NIHSS, National Institutes of Health Stroke Scale; ONT, onset-to-treatment time; SBP, systolic blood pressure.

*P < 0.05.

**TABLE 3:** Univariate logistic regression model for risk factors with hemorrhagic transformation (HT).

Variables	OR (95% CI)	*P*
**Demographics**		
Age	1.01 (0.99–1.04)	0.278
Female	0.88 (0.44–1.49)	0.494
**Medical history**		
Hypertension	1.41 (0.65–3.04)	0.383
Diabetes mellitus	0.81 (0.42–1.56)	0.530
Hyperlipidemia	0.85 (0.45–1.60)	0.614
Atrial fibrillation	2.86 (1.56–5.26)	0.001*
Coronary heart disease	2.05 (0.87–4.87)	0.102
**Previous medication use**		
Antiplatelets	1.56 (0.70–3.47)	0.281
**Cardiovascular risk factors**		
Current smoking	0.61 (0.27–1.35)	0.219
Current drinking	0.99 (0.47–2.10)	0.985
**Clinical features**		
NIHSS on admission	1.07 (1.03–1.10)	< 0.001*
Baseline SBP	1.01 (0.99–1.02)	0.554
Baseline DBP	1.01 (0.99–1.03)	0.224
Baseline blood glucose	1.01 (0.92–1.11)	0.814
WBC count	1.13 (1.04–1.22)	0.003*
Monocyte count	11.14 (3.22–38.50)	< 0.001*
Total triglycerides	0.76 (0.51–1.14)	0.180
Total cholesterol	0.85 (0.65–1.13)	0.261
HDL	1.06 (0.41–2.73)	0.91
MHR	6.41 (2.10–19.52)	0.001*
ONT	0.99 (0.98–1.02)	0.089
Platelet count	0.99 (0.98–1.01)	0.073
International normalized ratio	7.44 (0.61–90.92)	0.116
APTT	1.02 (0.98–1.06)	0.353
Bridge therapy	2.27 (1.03–5.03)	0.043*
**TOAST classification**		
Large–artery atherosclerosis	reference	-
Small–artery occlusion	1.74 (0.88–3.45)	0.112
Cardioembolic	0.31 (0.10–0.94)	0.038*
Other etiology	5.55 (0.33–92.41)	0.232
Undetermined etiology	0.73 (0.26–2.08)	0.556

APTT, activated partial thromboplastin time; DBP, diastolic blood pressure; HDL, high-density lipoprotein cholesterol; HT, hemorrhagic transformation; MHR, monocytes to high-density lipoprotein; NIHSS, National Institutes of Health Stroke Scale; ONT, onset-to-treatment time; SBP, systolic blood pressure.

*P < 0.05.

### Relationship between monocyte to high-density lipoprotein ratio and risk of hemorrhagic transformation

Median MHR was 0.44 (0.31–0.59) among all patients, and HT was associated with significantly higher MHR (0.53 vs. 0.42, *P* = 0.001). Multivariate logistic regression, after adjusting for potential confounders, identified MHR as an independent risk factor for HT. This result was obtained whether MHR was treated as a continuous variable (OR 7.50 per 1-SD increase, 95% CI 1.64–34.35, *P* = 0.009) or a quartile variable [Relative to the first quartile (< 0.31), OR 3.53 for third quartile (0.42–0.53), 95% CI 1.11–11.20, *P* = 0.032, OR 4.79 for fourth quartile (> 0.53), 95% CI 1.49–15.42, *P* = 0.009] (Table 4).

**TABLE 4:** Multivariate logistic regression to assess the potential relationship between monocyte to high-density lipoprotein ratio (MHR) and hemorrhagic transformation (HT).

Analysis	Model 1		Model 2	
	OR (95% CI)	*P*	OR (95% CI)	*P*
**MHR as continuous variable**				
Per 1-SD increase	4.22 (1.03–17.33)	0.046	7.50 (1.64–34.35)	0.009*
**MHR in quartiles**				
Quartile 1 (< 0.31)	1	–	1	–
Quartile 2 (0.31–0.42)	1.53 (0.44–5.29)	0.499	1.72 (0.49–6.01)	0.393
Quartile 3 (0.42–0.53)	2.88 (0.93–8.89)	0.066	3.53 (1.11–11.20)	0.032*
Quartile 4 (> 0.53)	3.36 (1.10–10.23)	0.033*	4.79 (1.49–15.42)	0.009*
NIHSS score	1.05 (1.01–1.09)	0.032*	1.05 (1.01–1.09)	0.023*
Atrial fibrillation	2.19 (1.09–4.38)	0.027*	2.48 (1.18–5.21)	0.017*

Model 1 was adjusted for atrial fibrillation, NIHSS score on admission, total triglycerides, international normalized ratio, bridge therapy, platelet count, and white blood cell count.

Model 2 was adjusted for the same variables as Model 1, as well as age, gender, diabetes mellitus, and baseline systolic blood pressure.

CI, confidence interval; NIHSS, National Institutes of Health Stroke Scale; OR, odds ratio.

*P < 0.05.

**FIGURE 2:**
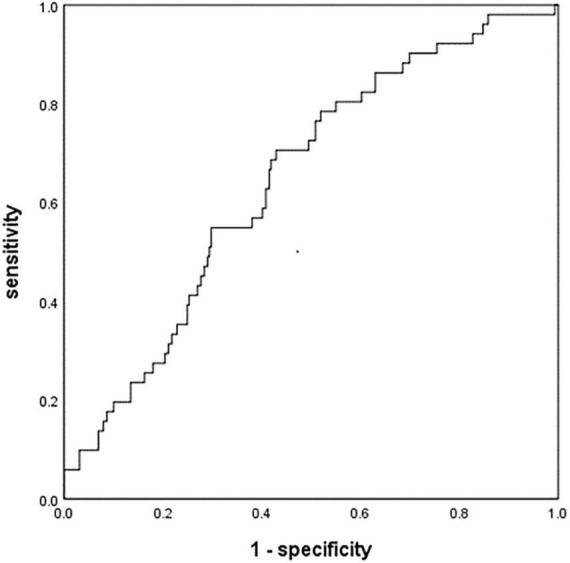
Receiver operating characteristic curve to assess the relationship between monocyte to high-density lipoprotein ratio (MHR) and hemorrhagic transformation (HT). AIS, acute ischemic stroke; HT, hemorrhagic transformation; MHR, monocyte to high-density lipoprotein ratio.

At the optimal cut-off of 0.46, MHR was able to differentiate between patients in our sample who experienced HT or not with a sensitivity of 70.6%, specificity of 57.1%, and AUC of 64.5% (95% CI 56.9–72.2%, *P* < 0.001; Figure 2).

## Discussion

The present study, one of few to investigate an association between MHR and HT in patients with acute ischemic stroke who receive intravenous thrombolysis, confirmed that higher MHR is associated with greater risk of HT. The ratio may therefore be useful for identifying at-risk patients and individualizing their treatment and management in order to optimize prognosis.

Our work extends the range of clinical contexts in which MHR serves as a useful inflammatory index for predicting prognosis. Previous work has demonstrated its ability to predict major adverse cardiac events and cardiovascular death in patients with coronary artery disease (Cetin et al., 2016; Karataş et al., 2016; Kundi et al., 2016; Wu et al., 2019; Jiang et al., 2022) and mortality in patients with acute pulmonary embolism (Avci et al., 2021). Previous studies on stroke patients have associated higher MHR with greater risk of poor 3-month functional outcome in patients with acute intracerebral hemorrhage (Liu et al., 2020), and with 30-day mortality among patients with acute ischemic stroke (Bolayir et al., 2018).

Our results confirm the usefulness of MHR as an index of post-stroke inflammation and associated secondary injury, including HT (Jickling et al., 2014). We previously showed that monocytes begin to infiltrate the ischemic area as early as 3 h after stroke onset (Choi et al., 2021), where they adhere to injured vascular endothelium (Tani et al., 2012). They migrate into the subendothelial space, where they mature into macrophages and internalize oxidized low-density lipoprotein, differentiating into foam cells (Kundi et al., 2016). These cells degrade tight junctions and release reactive oxygen species, cytokines and chemokines that disrupt cerebral microvascular endothelial cells, permeabilizing the blood-brain barrier (Canpolat, 2017). Monocytes also prolong immune responses to blood-brain barrier damage (Pedragosa et al., 2018; Liu et al., 2019). HDL counteracts the inflammatory and oxidative effects of monocytes on vascular endothelium by inhibiting their recruitment onto arterial walls, inhibiting oxidation of low-density lipoprotein and promoting cholesterol efflux from monocytes, preventing their differentiation into foam cells (Kundi et al., 2016). At the same time, HDL can recruit new endothelial cells to injured vascular endothelium, thereby promoting its repair (Riwanto et al., 2015).

Contrary to our findings, another study associated higher MHR with lower risk of HT in patients with ischemic stroke (Wang et al., 2020). It is possible that this discrepancy reflects the different functions of different monocyte subpopulations in the two studies. Computed tomography of our patients within 36 h after intravenous thrombolysis revealed infiltration of ischemic tissue by pro-inflammatory M1 monocytes. These monocytes contribute to adverse outcomes and death in patients with ischemic stroke (Urra et al., 2009). Infiltration in the previous study (Wang et al., 2020) may have involved primarily M2 monocytes, which secrete transforming growth factor-β1 and thereby promote collagen IV expression to maintain neurovascular integrity (Gliem et al., 2012). Future work should clarify the effects of different monocyte subpopulations on the blood-brain barrier in patients with acute ischemic stroke.

Hemorrhagic transformation occurred in 15.0% of our patients, which falls within the range of 10–43% reported in previous studies(Yaghi et al., 2017). We found that atrial fibrillation and higher NIHSS score were associated with greater risk of HT, confirming results from studies of reperfusion therapy (Yaghi et al., 2017).

Our findings should be interpreted with caution given that our sample came from a single center. Another limitation is that we did not monitor MHR dynamically, which might help clarify whether it is associated with HT. Future prospective studies should verify and extend our results.

## Conclusion

High MHR is independently associated with higher risk of HT among patients with acute ischemic stroke who receive intravenous thrombolysis. Thus, MHR may be a convenient, reliable marker to help identify at-risk patients and optimize treatment to avoid bleeding. Our study justifies further research into the complex effects of inflammation on HT and other post-stroke complications.

## Data availability statement

The original contributions presented in the study are included in the article/supplementary material, further inquiries can be directed to the corresponding author.

## Ethics statement

The studies involving human participants were reviewed and approved by Ethics Committee of the Second Affiliated Hospital and Yuying Children’s Hospital of Wenzhou Medical University. Written informed consent for participation was not required for this study in accordance with the national legislation and the institutional requirements. Written informed consent was obtained from the individual(s) for the publication of any potentially identifiable images or data included in this article.

## Author contributions

LX wrote the first draft of the article. All authors contributed to the proposal and design of the study as well as the collection, analysis, and interpretation of the data, and approved the submitted version.
